# Using Statistical Phylogenetics for Investigation of Enterovirus 71 Genotype A Reintroduction into Circulation

**DOI:** 10.3390/v11100895

**Published:** 2019-09-25

**Authors:** Yulia Vakulenko, Andrei Deviatkin, Alexander Lukashev

**Affiliations:** 1 Martsinovsky Institute of Medical Parasitology, Tropical and Vector Borne Diseases, Sechenov First Moscow State Medical University, 119435 Moscow, Russia; vjulia94@gmail.com; 2 Faculty of Biology, Lomonosov Moscow State University, 119234 Moscow, Russia; 3 Institute of Molecular Medicine, Sechenov First Moscow State Medical University, 119048 Moscow, Russia; andreideviatkin@gmail.com

**Keywords:** enterovirus, molecular epidemiology, enterovirus 71 (EV-A71), laboratory escape

## Abstract

Neurovirulent enterovirus 71 (EV-A71) caused a massive epidemic in China in 2008–2011. While subgenotype C4 was the major causative agent, a few isolates were almost identical to the prototype EV-A71 strain and belonged to genotype A. This variant was allegedly extinct since 1970, and its identification in this epidemic suggests reintroduction of the archive virus. Regression analysis of genetic distances (TempEst software) was of moderate utility due to the low resolution of classical phylogenetic methods. Bayesian phylogenetic analysis (BEAST software) suggested artificial introduction event based on highly aberrant phylogenetic tree branch rates that differed by over three standard deviations from the mean substitution rate for EV71. Manual nucleotide-level analysis was used to further explore the virus spread pattern after introduction into circulation. Upon reintroduction, the virus accumulated up to seven substitutions in VP1, most of them non-synonymous and located within the capsid’s canyon or at its rims, compatible with readaptation of a lab strain to natural circulation.

## 1. Introduction

Enterovirus 71 (EV-A71) belongs to the *Enterovirus* A species. This species, which also includes a number of Coxsackie A viruses and enteroviruses, is a member of the genus *Enterovirus* of the family *Picornaviridae*. EV-A71 is one of the main causative agents of hand, foot, and mouth disease (HFMD) and may cause severe neurological complications such as brainstem encephalitis and acute flaccid paralysis [1,2]. In 2008–2011, regular EV-A71 epidemics that involved over a million cases occurred in China [3]. At present, EV-A71 is the most represented enterovirus type in GenBank, with over 11,000 partial genomic sequences out of approximately 70,000 enterovirus sequences [4]. According to the genetic distance and phylogenetic relations in the genome region that encodes the major capsid protein VP1, EV-A71 is classified into six genotypes and 14 subgenotypes (A, B1–5, C1–5, D, E, and F) [1,5]. Genotypes B and C dominate in circulation globally [1]. In particular, the Chinese EV-A71 epidemic was caused by subgenotype C4 [6]. Genotypes D, E, and F were found only in a few limited areas, namely Central and West Africa, India, and Madagascar [5,7,8,9]. Genotype A has been represented by a sole prototype strain (BrCr), which was identified in California, USA, during 1969–1972 [10]. Although the most recent cases in China were associated with subgenotype C4, several isolates belonged to genotype A, which had not been detected since 1972 [11,12,13]. The high degree of identity between the Chinese EV-A71 GtA and the prototype strain BrCr was suggestive of non-natural introduction [5]. However, the possible release and spread of strain BrCr in China has not been analyzed in detail. Here, we used Bayesian phylogenetic analysis to investigate the reintroduction of EV-A71 GtA in China.

## 2. Materials and Methods

To generate the alignment of full EV-A71 VP1 sequences, all available EV-A71 sequences (11,200 by January 2018) were obtained from GenBank. Sequences less than 800 nucleotides (nt) and over 8000 nt were omitted. The sequences that overlapped with the VP1 region in prototype strain BrCr (GenBank accession JN874547) were identified using blastn search [14]. The sequences that contained the VP1 region were aligned using MAFFT v.7.304 [15]. The full VP1 sequence (891 nt) was excised from the alignment according to the VP1 sequence of the prototype strain BrCr. Then, redundant sequences that shared more than 95.5% overall nucleotide sequence identity with any other sequence in the alignment were removed. Data with apparent sequencing errors (e.g., frameshifts or unnatural heterogeneity at sequence termini) were excluded manually. The resulting alignment contained 97 full VP1 sequences representative of the entire known EV-A71 VP1 gene diversity. Blastn search of BrCr VP1 sequence against the GenBank database yielded 18 Chinese isolates from 2008–2011 with a VP1 region almost identical (>99%) to the BrCr strain. Sixteen of these sequences were added to the alignment of EV-A71 full VP1 gene sequences, while two (GQ117124 and GQ117126) were omitted due to potential sequencing errors. The Python scripts used to generate the alignment are available on (GitHub https://github.com/v-julia/GenAlignment).

The Kimura 2-parameter model with invariable sites was the best-fit substitution model under the Akaike information criterion, as determined with MEGA7 software [16]. Maximum likelihood (ML) phylogenetic trees under the Kimura 2-parameter model with invariable sites were built for the alignment. It comprised 113 representative EV-A71 VP1 gene sequences, including 16 BrCr-like Chinese sequences. This tree was rooted at the midpoint and was further analyzed using TempEst (formerly Path-O-Gene) software [17] to assess the temporal structure of the data set. Also, ML trees were built for the sole GtA (4 non-identical BrCr prototype strains and 16 Chinese BrCr-like sequences) and the sole GtC was represented by 55 sequences from the initial alignment of EV-A71 with 95.5% identity cutoff. The phylogenetic trees of the sole GtA and GtC were analyzed in TempEst using the best-fitting root function.

Bayesian phylogenetic analysis of the EV-A71 VP1 sequence alignment (*N* = 113) and an alignment that included only 16 EV-A71 GtA sequences reported in China in 2008–2011 was performed in the Bayesian statistical framework using the Markov chain Monte Carlo (MCMC) approach implemented in BEAST 1.10.4 [18]. SRD06 model (a modification of the HKY substitution model with gamma-heterogeneity of rates and partitioning of sequence data into (1^st^ + 2^nd^) and 3^rd^ codon positions [19]) was used with the uncorrelated relaxed log-normal molecular clock assumption [20] and constant population size. These model parameters were previously shown to be preferential for enterovirus datasets [21]. The analyses were run for 1.00 × 10^8^ and 5.00 × 10^6^ generations for all EV-A71 and the sole GtA, respectively; trees were sampled every 10,000 generations. Convergence and mixing properties were inspected using Tracer 1.6 [22]. Maximum clade credibility (MCC) trees were annotated with TreeAnnotator v.1.10.8 using a burn-in of 2.00 × 10^7^ and 5 × 10^5^ generations, respectively. Trees were visualized with FigTree v.1.4.2 [23]. Branch substitution rates were extracted from the MCC tree file, and their frequency distribution histograms were plotted using Python scripts (https://github.com/v-julia/sample_bias).

The mutations in the Chinese EV-A71 GtA sequences were inspected manually using BioEdit [24]. In addition to JN874547, three other non-identical sequences of the prototype strain BrCr that originated from different laboratories (AB204852, AB204853, and U22521) were added to the alignment. 

## 3. Results

**Figure 1:**
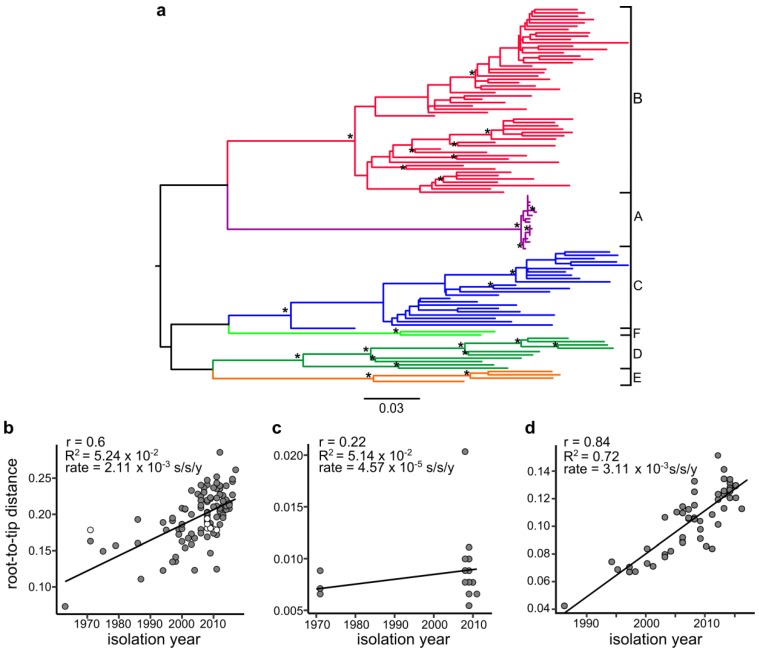
(**a**) Maximum likelihood (ML) phylogenetic tree used for TempEst analysis (panel (**a**)) inferred under a Kimura 2-parameter model with invariable sites. Stars at tree nodes indicate nodes with bootstrap values greater than 80%. Letters to the right and branch colors indicate enterovirus 71 (EV71) genotypes. TempEst analysis of the temporal population structure in the total EV-A71 alignment with 16 BrCr-like Chinese sequences, midpoint tree root (**b**), the sole genotype A (16 Chinese sequences and 4 prototype BrCr sequences), best-fitting tree root (**c**), and the sole genotype C, represented by 55 sequences (**d**). White circles in panel (**b**) correspond to genotype A sequences (prototype strain sequence JN874547 and Chinese sequences), and grey circles correspond to the sequences of other genotypes. The correlation coefficient, *R*^2^, and substitution rate (regression line slope) are indicated in each plot.

Reintroduction of EV-A71 genotype A into circulation in China in 2008 was previously suspected [5] but was neither proven statistically nor investigated in detail. Phylogenetic methods can verify virus reintroduction by assaying temporal structure in the virus population. This could be conducted in the case of the positive correlation between tree root-to-tip distance and tree tip isolation times. Biological properties of enteroviruses make them generally well suited for such investigations, because they accumulate substitutions at high rates, usually between 0.4–1.2 × 10^−2^ substitutions/site/year (s/s/y) [21]. They are also unlikely to persist in a host or be preserved in natural settings, factors that could cause major disturbance of evolutionary estimates of individual viruses. Thus, we created an alignment that comprised all EV-A71 sequences downloaded from GenBank (as of January 2018) that shared less than 95.5% sequence identity (*N* = 97, 891 nt). Sixteen Chinese BrCr-like sequences were manually added to this alignment. First, a ML phylogenetic tree was built (Figure 1a) and the dataset was tested by the TempEst software to analyze whether it had a temporal structure and was thus suitable for molecular clock analysis.

TempEst indicated a positive correlation between isolation time and distance from tree root to tip (sum of all branch lengths from the root to a tip) for the VP1 structural-protein-encoding sequences (Figure 1b, correlation coefficient = 0.6, *R*^2^ = 0.36). Given the high substitution rate of enteroviruses (0.4–1.2 × 10^−2^ substitutions per site per year), the nucleotide distance of 0.1–0.8 × 10^−2^ between the prototype BrCr strain and Chinese sequences was unlikely to be maintained upon 40 years of natural circulation. However, the Chinese isolates did not significantly deviate from the regression line upon visual inspection (white circles, Figure 1b). Analysis of only EV-A71 GtA (4 BrCr prototype strains and 16 Chinese BrCr-like sequences) using TempEst revealed a weak positive correlation (Figure 1c, correlation coefficient = 0.22, *R*^2^ = 5.14 × 10^−2^) between isolation time and distance from a hypothetical tree root to tip. This observation questioned the temporal structure of the EV-A71 GtA population because the correlation between tip dates and branch lengths from the tree root was very weak. This was strikingly different from a strong correlation observed, for example, for EV-A71 GtC (Figure 1d, correlation coefficient = 0.84, *R*^2^ = 0.72). The substitution rate estimated by regression line slope for the whole EV-A71 (Figure 1b, 2.11 × 10^−3^ s/s/y) was roughly twice slower than typical EV-A71 substitution rates inferred by Bayesian phylogenetics (4–6 × 10^−3^ s/s/y [21,25]), which is reasonable given the profound difference between these methodologies. The substitution rate for EV-A71 GtA (Figure 1c, 4.57 × 10^−5^ s/s/y), however, was 46 times lower than for the whole EV-A71 (Figure 1b) and 68 times lower than for EV-A71 GtC (Figure 1d, 3.11 × 10^−3^ s/s/y). Such rate variations are not observed in nature and, therefore, the results of regression analysis were compatible with a non-natural evolution of the Chinese GtA viruses. However, the results of regression analysis neither provided statistical support for the findings nor allowed further investigation of the virus spread.

Another hint to the artificial origin of GtA in China came from the recombination analysis. Only one Chinese GtA complete genome sequence was available (KF501389). There was no evidence of recombination relative to the prototype BrCr sequence. Even though EV-A71 has a distinctively low recombination rate among other enteroviruses, the median half-life of a non-recombinant node is 13–18 years [26], and circulation of a non-recombinant virus for 38 years does not seem likely.

**Figure 2:**
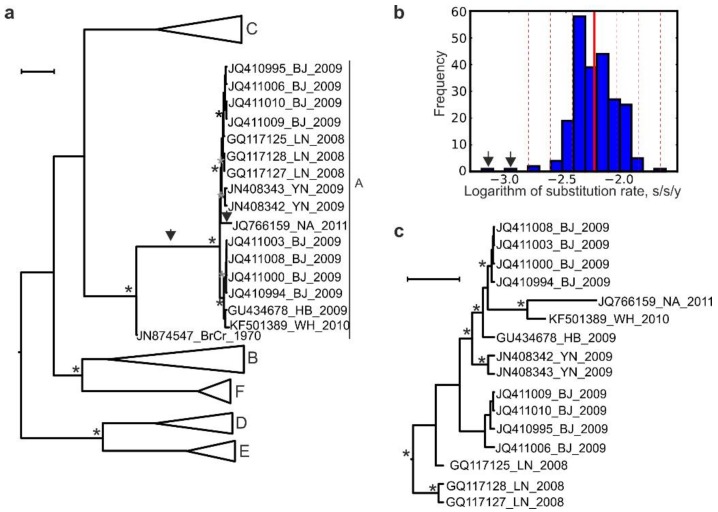
(**a**) Maximum clade credibility (MCC) tree for EV-A71 sequences of different subtypes and 16 BrCr-like Chinese isolates. The scale bar below the tree corresponds to 10 years. Subtypes other than A are collapsed for graphical reasons. The asterisks indicate node posterior probabilities above 0.85. (**b**) Frequency distribution of branch substitution rate logarithms in the MCC tree shown in panel (**a**). The solid red line indicates the mean, and the dashed lines indicate standard deviations from the mean. (**c**) MCC tree for 16 BrCr-like Chinese sequences. The scale bar below the tree corresponds to one year. Abbreviations in Chinese sequences’ names correspond to the cities or provinces of isolation: BJ—Beijing, HB—Hubei province, LN—Luan, WH—Wuhan, YN—Yunnan, NA—unknown.

Bayesian phylogenetic methods use virus isolation times (tree tip dates) as an additional parameter in inferring a phylogenetic tree that better reflects the actual virus evolution. Bayesian phylogenetic analysis of the full VP1 sequences (891 nt) from EV-A71 strains that represented all genotypes and differed by at least 4.5% in the nucleotide sequence (*N* = 97) and 16 GtA sequences reported in China in 2008–2011 was performed using BEAST v.1.10.4 (Figure 2a). The mean substitution rate in the resulting phylogenetic tree was 5.7 × 10^−3^ s/s/y, or −2.22 log s/s/y, which corresponds to most previous estimates for EV-A71 [5,21,25,27]. Substitution rates for most phylogenetic tree branches within the Chinese GtA group did not deviate from the mean rate of the tree by more than three standard deviations, with two exceptions (Figure 2a, arrows). The branch from the prototype strain BrCr (USA, 1970) to the common ancestor of the Chinese GtA isolates had a rate of 1.00 × 10^−3^ s/s/y (−2.98 log s/s/y), and the terminal branch to the Chinese isolate JQ766159 (2011) had a rate of 6.00 × 10^−4^ s/s/y (−3.22 log), 5.7 and 9.5 times lower than the mean rate for EV-A71, respectively. These branch rate values differed by over three standard deviations from the mean rate for EV-A71 (Figure 2b, arrows). This result is compatible with a non-natural introduction of the Chinese GtA strains. The most recent common ancestor (MRCA) of the Chinese GtA sequences was inferred in 2005 (95% highest posterior density (HPD) interval, 2000–2010) on a tree that included other EV-A71 sequences (Figure 2a). A separate analysis of the Chinese GtA isolates without laboratory BrCr strains and other EV-A71 genotypes confirmed the MRCA date in 2005 (95% HPD interval, 2003–2008; Figure 2c), while the substitution rates across this tree were also within typical EV-A71 values (4.0 × 10^−3^ to 7.0 × 10^−3^ s/s/y). This result suggests “normal” circulation of GtA in China upon its putative introduction. However, the aberrant rate of its ancestral branch (Figure 2a, arrow) raised concerns regarding the validity of the phylogenetic inference within that group and called for a detailed manual analysis of GtA spread in China.

First, an exact source of GtA introduction needed to be traced. Enteroviruses accumulate mutations upon passaging in cell culture, and there are four sequences of the prototype strain BrCr in GenBank (accession numbers: JN874547, AB204852, AB204853, and U22521); they differ by up to 11 substitutions in the VP1 genome region. Two prominent polymorphisms among these sequences differentiated the laboratory strain BrCr variants from each other: C/A in VP1 nucleotide position 724 and H/Y in VP1 amino acid position 116 (Table 1).

One GtA isolate (sequence JQ766159) was obtained in 2011 in China (collection city is not indicated) from a throat swab and had a rare nucleotide substitution (724C), which did not occur in any of 7026 EV-A71 full VP1 gene sequences except for two prototype strain BrCr sequences (AB204852 and AB204853) and two unrelated subgenotype C1 sequences. This mutation was also absent from all of the remaining Chinese GtA sequences (Table 1). This finding suggests that “isolate” JQ766159 could be a laboratory contamination by an AB204853-related BrCr lineage, because there is no evidence that this virus variant actually circulated. Importantly, sequence JQ766159 branch had an abnormally slow substitution rate upon Bayesian reconstruction (see above), concordant with a phylogenetic history independent from other Chinese GtA sequences.

**Figure 3:**
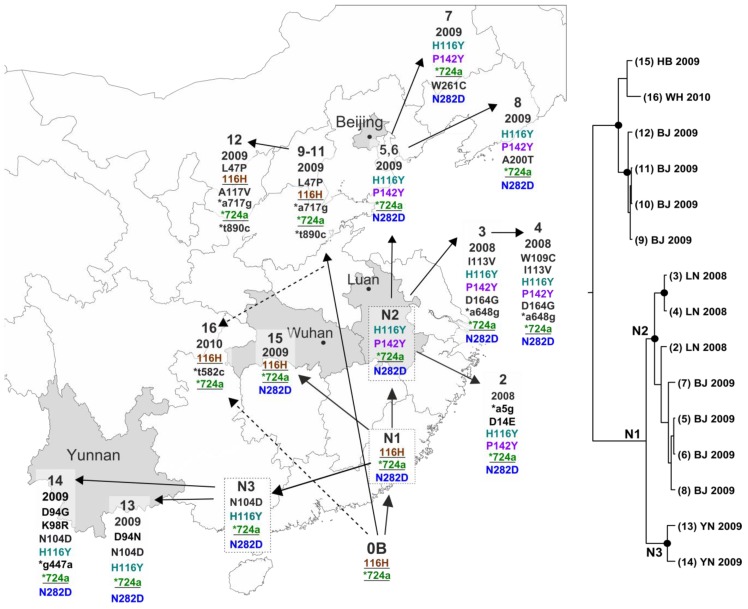
Possible pattern of EV-A71 genotype A release and spread in China. Hypothetical intermediate ancestors of present viruses are indicated in dashed squares. Suggestive source strain is 0B (Table 1). Isolates are labeled with Arabic numbers, hypothetical intermediate viruses with N1–N3. Arrows mark likely transmission events and dashed arrows indicate alternative transmission events. Isolation year and substitutions in the VP1 region are provided. Synonymous nucleotide mutations are given in small letters and marked with asterisks and amino acid substitutions are in capital letters; positions are given for VP1-encoding sequence or the protein, respectively. Key mutations mentioned in the text are shown in color. Polymorphisms that likely originated from the heterogeneity observed among source BrCr variants are underlined. Full information about sequences is provided in Table 1. Provinces where BrCr-like Chinese viruses were isolated are colored in grey. The MCC phylogenetic tree was built using Chinese GtA sequences and BrCr sequence JN874547, which was used to root the tree, but then omitted. Tips are numbered according to the map. Nodes with posterior probabilities above 0.9 are labeled with dots. Nodes that correspond to the putative ancestors are labeled. The map was downloaded from https://simplemaps.com/resources/svg-cn.

All other Chinese GtA isolates had adenosine at VP1 position 724, which is common among EV-A71 sequences. However, some of them had another distinctive amino acid substitution 116H. This substitution was only found in two BrCr strain sequences (AB204853, sequenced in Japan in 2005 [28], and JN874547, sequenced in Taiwan in 2011 [29]) and in 7 of 16 Chinese GtA isolates. It was not found in any of the 7026 available EV-A71 sequences of all genotypes and favors common origin of Chinese GtA isolates (Table 1, sequences 2–16) from a single introduction of a virus that was most related to the BrCr strain sequence JN874547 (Figure 3, 0B). A single introduction event would also be compatible with an aberrant substitution rate of the branch leading from the archive strain BrCr to all Chinese GtA sequences (Figure 2a). 

All the remaining Chinese GtA isolates had up to seven additional mutations in VP1 compared to JN874547, the most likely sequence of origin (Table 1). Most diversification events could be inferred with relative certainty, but in one case there were two possible patterns (Figure 3, dashed lines). 

Upon introduction, the virus diverged into two or three lineages. One of them was detected in Beijing in 2009 [13] (Figure 3, sequences 9–12) and had one additional amino acid and two synonymous nucleotide substitutions. Another lineage spread all over Southeast China; its typical feature was an N282D substitution, which is very rare among other EV-A71 sequences (33 sequences out of 7026) and supports a common origin for this group. In some of these viruses, the 116H amino acid reverted to the 116Y variant, which is very common among circulating EV-A71. Presence of a very rare N282D substitution in all 116Y sequences supports the aforementioned sequence of events and thus a single contamination event rather than introduction of two distinct virus variants, one with 116H and another with 116Y polymorphisms. The major N282D lineage included two hypothetical intermediate strains (Figure 3, N1 and N2), which further diverged into lineages found in Luan (Anhui province) in 2008 (sequences 2–4), the Yunnan province 2009 lineage (N3 ancestor and sequences 13 and 14), the Beijing 2009 lineage (Figure 3, sequences 5–8) [13], and the Hubei province 2009–2010 lineage (Figure 3, sequence 15) [11]. These lineages carried unique characteristic mutations and gained additional substitutions during circulation. The origin of sequence 16 remains uncertain, because it could result from a direct spread of the original virus to Hubei province, from a common ancestor with Beijing sequences, or from an independent introduction. Phylogenetic analysis was generally compatible with this sequence of events (Figure 3). Putative intermediate sequences N1, N2, and N3 corresponded to distinct tree nodes. However, there was one notable exception. Phylogenetic analysis placed sequence 15 together with isolates 9–12 and 16. This topology could be justified by the number of substitutions, but knowing that the N282D mutation is very rare in EV-A71, it appears more parsimonious that it emerged just once in the putative ancestor N1.

**Figure 4:**
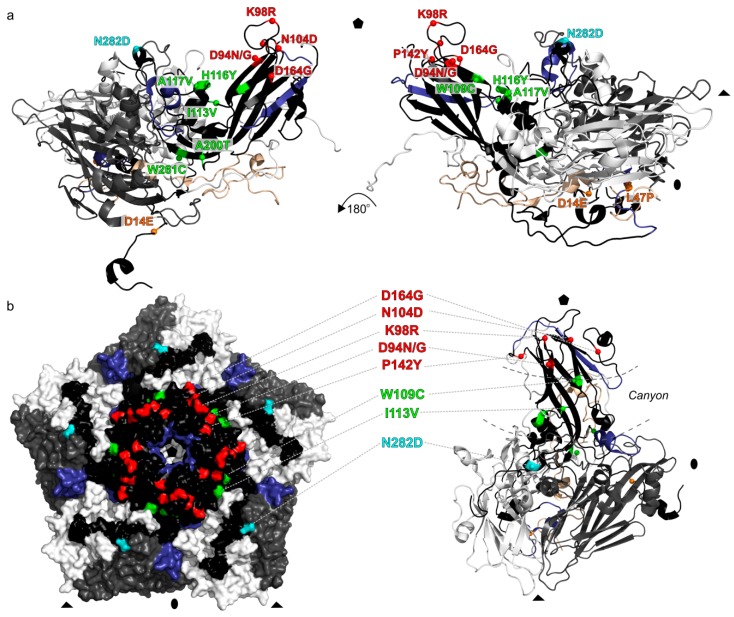
Mapping of 14 amino acid substitutions found in Chinese BrCr-like isolates. The residues are colored according to the structural region of the capsid: the canyon northern rim (red), canyon floor and its proximity (green), canyon southern rim (cyan), and other regions (orange), based on the EV-A71 structure 4CDQ [36]. Residues that belong to the major epitopes of VP1 [34] are colored in dark blue. (**a**) Side views of a cartoon representation of the EV-A71 icosahedral asymmetric unit; (**b**) surface residues mapped to the molecular surface of EV-A71 pentamer formed by five icosahedral asymmetric units (left) and a single icosahedral asymmetric unit in cartoon representation (right) viewed down a five-fold axis. The capsid VP1 protein is colored in black, VP2 colored in grey, VP3 colored in white, and VP4 colored in wheat. Positions of the icosahedral symmetry elements are indicated. The visualization of EV-A71 crystal structure was performed in PyMOL [37].

It is noteworthy that most of the substitutions among the circulating GtA strains were non-synonymous. The usual ratio of non-synonymous to synonymous substitutions in enterovirus structural genes is between 0.029 and 0.172 [25]; it was 0.04 (or 1 non-synonymous per 25 synonymous substitutions) in the EV-A71 VP1 dataset used here (all 7026 sequences), but as high as 1.33 among the circulating GtA sequences. This observation could be compatible with readaptation of a cell-culture-adapted virus to circulation among humans. Indeed, mapping of the 14 amino acid substitutions on the capsid surface showed that 12 of them were located in the canyon or at its rims (Figure 4), regions that participate in binding with receptors and antibodies [30,31,32,33,34] and commonly harbor immune escape mutations [35]. In particular, N282D substitution was reported to be responsible for a neutralization escape [35]. This mutation was found in just 33 of 7026 circulating EV-A71 isolates but occurred in the common ancestor N1 early in the history of GtA circulation in China and could have been pivotal for its further spread.

## 4. Discussion

Development of synthetic biology and bioterrorism threats require statistical tools for investigation of potential non-natural events in virus evolution. Root-to-tip distance regression analysis is a method of choice for such analysis and has been successfully used previously [38,39]. In the investigation of Venezuelan equine encephalitis introduction, it was suggested to use the regression line slope to infer nucleotide sequence change rates and thus non-natural evolutionary events in certain sequence groups [39]; however, this parameter may be dependent upon the dataset and is difficult to evaluate statistically, and also requires that there is a group of “artificial” sequences, which may not be always available.

The introduction of GtA into circulation was highly likely from examining genetic distances between the BrCr strain and Chinese GtA isolates, which were less than 1%. Approximately 20% of substitutions could be expected given the 38-year interval and ca. 0.5%/year substitution rate in EV-A71. Regression analysis of the whole EV-A71 dataset did not reveal abnormalities in the Chinese GtA sequences. The failure of regression analysis in this case can be explained by mutation saturation close to the EV-A71 tree root. Indeed, all EV-A71 sequences differed by up to 29% in the nt sequence (with 0.04 dN/dS ratio), which roughly corresponds to the exploration of all available synonymous sites. On the other hand, EV-A71 accumulates 0.5% substitutions/year, so reverse substitutions could impact phylogenetic analysis on a scale of a few decades, while the estimates of the most recent common EV-A71 ancestor date back 67–80 years [5,21,25]. Concordant with this hypothesis, analysis of distinct EV-A71 genotype C using regression analysis produced a higher correlation coefficient and more homogeneous distribution of root-to-tip distances around the correlation curve than observed for the whole type (Figure 1d). The substitution rate inferred for GtA upon regression analysis (Figure 1c) was compatible with aberrant evolutionary history; however, a more detailed investigation called for additional methods.

Bayesian statistical phylogenetics used isolation year data and yielded a well-resolved phylogenetic tree that allowed detecting suspicious evolutionary events among EV-A71 GtA isolates. Branch rates proved to be a useful tool for identifying such events, while extracting all rates from a phylogenetic tree and plotting their distribution provided a statistical measure of observed deviations. In the particular case studied here, a difference of four to five standard deviations from the mean corresponded to *p*-values well below 0.001. Importantly, this approach may not be applicable in all cases. Statistical phylogenetic algorithms aim at providing the most parsimonious image of natural evolution according to the selected evolution model. This design may mask less obvious non-natural events by smearing aberrant substitution rates between several neighboring branches. Additionally, even though branch rate analysis provided some sort of statistical support for the findings, it should be treated with caution, because it compares inferred values and not real-life observations. On the other hand, root-to-tip regression analysis is also recommended only for exploratory analysis and does not allow for formal hypothesis testing [17].

In simulated datasets, detection of abnormal tip dates shifted by five years was unlikely, while shifts of 20 years were usually detectable using the approach described above [40]. On the other hand, a small fraction of GenBank entries may indicate wrong collection dates. Therefore, not all divergent branch rates are a result of virus introduction but may represent experimental artifacts. Additional analysis was performed here to further prove that EV-A71 GtA emergence in China was a virus release but not a series of lab contaminations.

Bayeisan phylogenetic analysis yielded a substitution rate of 4.60 × 10^−3^ s/s/y among the Chinese GtA isolates, which is typical to natural circulation of EV-A71. A relatively short VP1 genome region (891 nt) may lack resolution on a short time scale [4], complicating a phylogenetic study. Manual point mutation analysis suggested a complex pattern of virus circulation in China. Importantly, the ratio of non-synonymous to synonymous substitutions in circulating GtA (1.33) was much higher than in EV-A71 in general (0.04). This observation may correspond to readaptation of a cell-culture-adapted lab strain to circulation in the human population, or adaptation of a virus of USA origin to the Chinese population. Indeed, 12 of 14 amino acid substitutions mapped within or at a close proximity of the known receptor and antibody binding sites. Further, this suggests that the evolutionary patterns in the Chinese GtA lineage could differ from those in normally circulating EV-A71. Noteworthy, a similar rapid accumulation of non-synonymous substitutions was observed in attenuated lab-passaged poliovirus strains upon administration of the live vaccine to humans [41]. The dating of the MRCA of the circulating Chinese GtA (2005) should be treated with caution, because the isolation dates of most viruses only contained the year, limiting precise short-scale analysis, and rapid readaptation of the virus to natural circulation could disturb the molecular clock analysis. Common sense suggests that virus release was more likely in 2008, upon the onset of the epidemic and the corresponding studies in virological laboratories, which routinely use prototype strains as positive controls. The latter introduction date fits within the 95% confidence interval of our Bayesian analyses.

EV-A71 GtA viruses have been isolated in four provinces of China from 2008–2010. Apparently, the scale of virus circulation was extensive. All 15 isolates analyzed here came from hand, foot, and mouth disease (HFMD) patients, none of whom had neurological disease [11,12,13]. This small number of isolates does not allow ruling out neuropathogenicity of the Chinese GtA viruses. However, BrCr-derived strains are clearly capable of causing HFMD, while no such symptoms were reported for the original strain BrCr in the USA [10]. Similarly, other genotypes of EV-A71 circulate widely in the world, but do not cause such massive HFMD outbreaks as in China and Southeast Asia [1,42]. Isolation of GtA from HFMD patients further supports a general view that host genetics or local factors rather than an intrinsic virulence of EV-A71 drove the Asian EV-A71 epidemic.

The last published sequence of BrCr-like EV-A71 dates to 2011. The virus has not been reported outside of China, nor implicated in significant disease outbreaks. This fact could correspond to lower pathogenicity of strain BrCr in vivo compared to EV-A71 genotype B [43], or it could reflect a general emergence–extinction pattern typical of enterovirus circulation [44].

The case of EV-A71 GtA reintroduction indicates that virus escape from laboratories remains a realistic threat. It adds to the history of well-known releases of the H1N1 influenza virus in 1977 [39,45] and smallpox in the UK [46], as well as many more less famous release events [47].

## Figures and Tables

**Table 1:** Nucleotide and amino acid substitutions in BrCr-like Chinese sequences.

Figure 3 ID	GenBank Accession	Location	Year	Reference	Synonymous Nucleotide Substitutions							Amino Acid Substitutions													
					5	447	582	648	717	724	890	14	47	94	98	104	109	113	116	117	142	164	200	261	282
0A	AB204853	*	-	[28]	a	g	t	a	a	c	t	D	L	D	K	N	W	I	H	A	P	D	A	W	N
1	JQ766159	Unknown	2011	D.s.	-	-	-	-	-	-	-	-	-	-	-	-	-	-	-	-	-	-	-	-	-
0B	JN874547	the USA	1970	[29]	a	g	t	a	a	a	t	D	L	D	K	N	W	I	H	A	P	D	A	W	N
N1 **	-	-	-	-	-	-	-	-	-	-	-	-	-	-	-	-	-	-	-	-	-	-	-	-	D
N2 **	-	-	-	-	-	-	-	-	-	-	-	-	-	-	-	-	-	-	-	-	Y	-	-	-	D
2	GQ117125	Luan	2008	[12]	g	-	-	-	-	-	-	E	-	-	-	-	-	-	Y	-	Y	-	-	-	D
3	GQ117128	Luan	2008	[12]	-	-	-	g	-	-	-	-	-	-	-	-	-	V	Y	-	Y	G	-	-	D
4	GQ117127	Luan	2008	[12]	-	-	-	g	-	-	-	-	-	-	-	-	C	V	Y	-	Y	G	-	-	D
5	JQ411010	Beijing	2009	[13]	-	-	-	-	-	-	-	-	-	-	-	-	-	-	Y	-	Y	-	-	-	D
6	JQ411009	Beijing	2009	[13]	-	-	-	-	-	-	-	-	-	-	-	-	-	-	Y	-	Y	-	-	-	D
7	JQ411006	Beijing	2009	[13]	-	-	-	-	-	-	-	-	-	-	-	-	-	-	Y	-	Y	-	-	C	D
8	JQ410995	Beijing	2009	[13]	-	-	-	-	-	-	-	-	-	-	-	-	-	-	Y	-	Y	-	T	-	D
9	JQ411003	Beijing	2009	[13]	-	-	-	-	g	-	c	-	P	-	-	-	-	-	-	-	-	-	-	-	-
10	JQ411000	Beijing	2009	[13]	-	-	-	-	g	-	c	-	P	-	-	-	-	-	-	-	-	-	-	-	-
11	JQ411008	Beijing	2009	[13]	-	-	-	-	g	-	c	-	P	-	-	-	-	-	-	-	-	-	-	-	-
12	JQ410994	Beijing	2009	[13]	-	-	-	-	g	-	c	-	P	-	-	-	-	-	-	V	-	-	-	-	-
15	GU434678	Hubei province	2009	D.s	-	-	-	-	-	-	-	-	-	-	-	-	-	-	-	-	-	-	-	-	D
16	KF501389	Wuhan	2010	[11]	-	-	c	-	-	-	-	-	-	-	-	-	-	-	-	-	-	-	-	-	-
N3 **	-	-	-	-	-	-	-	-	-	-	-	-	-	-	-	-	-	-	Y	-	-	-	-	-	D
13	JN408342	Yunnan	2009	D.s.	-	-	-	-	-	-	-	-	-	N	-	D	-	-	Y	-	-	-	-	-	D
14	JN408343	Yunnan	2009	D.s.	-	a	-	-	-	-	-	-	-	G	R	D	-	-	Y	-	-	-	-	-	D

* A laboratory strain with a significant passaging history derived from the original strain BrCr, see [28]. ** Hypothetical intermediate viruses. D.s.—direct submission. Dashes indicate the nucleotides and amino acids that are identical to the the reference sequences (0A or 0B).
